# P-2184. Challenges of dengue in haematological malignancy patients: Analysis from the DANGO registry during the 2023-2024 outbreak in Argentina

**DOI:** 10.1093/ofid/ofaf695.2347

**Published:** 2026-01-11

**Authors:** Jon Salmanton-Garcia, Carla Niveyro, Pedro A Villalba apestegui, Victoria Isabel Martin, Maria Cynthia Tomasino, Mariana Del Carmen Marull, Claudia Patricia Lorena Fernandez, Fernanda Tosin, Maria Fernanda Hobecker, Karen Batrice Duranona, Angela Daiana Jara, Oliver A Cornely, Gustavo-Adolfo Méndez

**Affiliations:** University Hospital Cologne, Cologne, Germany, Cologne, Nordrhein-Westfalen, Germany; Madariaga Hospital, Posadas, Misiones, Argentina; Infectious Diseases Department, Hospital Escuela de Agudos Dr. Ramón Madariaga. Posadas, Misiones – Argentina, posadas, Misiones, Argentina; 1. Hospital Escuela de Agudos Dr Ramón Madariaga, Posadas, Misiones, Argentina, posadas, Misiones, Argentina; 1. Hospital Escuela de Agudos Dr Ramón Madariaga, Posadas, Misiones, Argentina, posadas, Misiones, Argentina; 1. Hospital Escuela de Agudos Dr Ramón Madariaga, Posadas, Misiones, Argentina, posadas, Misiones, Argentina; 1. Hospital Escuela de Agudos Dr Ramón Madariaga, Posadas, Misiones, Argentina, posadas, Misiones, Argentina; 1. Hospital Escuela de Agudos Dr Ramón Madariaga, Posadas, Misiones, Argentina, posadas, Misiones, Argentina; 1. Hospital Escuela de Agudos Dr Ramón Madariaga, Posadas, Misiones, Argentina, posadas, Misiones, Argentina; 1. Hospital Escuela de Agudos Dr Ramón Madariaga, Posadas, Misiones, Argentina, posadas, Misiones, Argentina; 1. Hospital Escuela de Agudos Dr Ramón Madariaga, Posadas, Misiones, Argentina, posadas, Misiones, Argentina; University of Cologne, Faculty of Medicine and University Hospital Cologne, Cologne, Nordrhein-Westfalen, Germany; Hospital Escuela de Agudos Dr Ramón Madariaga, Posadas, Misiones, Argentina, Posadas, Misiones, Argentina

## Abstract

**Background:**

Dengue is a single-stranded RNA virus with four serotypes, transmitted primarily through the *Aedes aegypti* mosquito. Haematological malignancy (HM) patients are at heightened risk for severe dengue due to their immunocompromised state, yet the infection is often underdiagnosed in this group due to overlapping symptoms with their underlying condition or treatments.

**Methods:**

This retrospective multicentre study analysed HM patients diagnosed with dengue between November 2023 and May 2024. Using data from the DANGO registry, which documented cases from seven hospitals in Argentina during the dengue epidemic 2023-2024, the study included patients aged ≥ 16 years with confirmed dengue and existing HM, collecting data on clinical presentation, laboratory findings, and outcomes.

**Results:**

The study included 33 HM patients diagnosed with dengue. The median age was 54 years. The most common comorbidities were hypertension (18%) and renal insufficiency (9%), and the most frequent HM was non-Hodgkin lymphoma (30%). Laboratory findings indicated severe/very severe thrombocytopenia (52%) and leukopenia (67%) in the majority of patients. The study found that 64% of patients required hospitalization, for a median duration of 7 days. The overall mortality rate was 6%, with all deaths associated with dengue complications.

**Conclusion:**

Dengue in HM patients poses diagnostic challenges due to symptom overlap with other conditions. This study emphasizes the need to consider dengue in febrile illnesses during outbreaks in endemic areas, advocating for early diagnosis, adequate management, and possibly routine DENV testing in this population to reduce morbidity and mortality.

**Disclosures:**

Jon Salmanton-Garcia, MSc, MPH, PhD, menarini, gilead, astrazeneca, pfizer: Honoraria Oliver A. Cornely, Prof. Dr., Al-Jazeera Pharmaceuticals/Hikma: Honoraria|Basilea: Advisor/Consultant|Cidara: Advisor/Consultant|Cidara: Board Member|Cidara: Grant/Research Support|Elion: Advisor/Consultant|F2G: Grant/Research Support|Gilead: Advisor/Consultant|Gilead: Grant/Research Support|Gilead: Honoraria|GlaxoSmithKline: Advisor/Consultant|GlaxoSmithKline: Honoraria|Grupo Biotoscana/United Medical/Knight: Honoraria|Melinta: Advisor/Consultant|Melinta: Board Member|MSD: Honoraria|Mundipharma: Advisor/Consultant|Mundipharma: Grant/Research Support|Mundipharma: Honoraria|Pfizer: Advisor/Consultant|Pfizer: Grant/Research Support|Pfizer: Honoraria|Pulmocide: Board Member|Scynexis: Advisor/Consultant|Scynexis: Grant/Research Support|Shionogi: Advisor/Consultant|Shionogi: Honoraria

